# Childhood dental fear in children aged 7-11 years old by using the Children’s Fear Survey Schedule-Dental Subscale

**DOI:** 10.25122/jml-2020-0084

**Published:** 2021

**Authors:** Sujata Rath, Debasmita Das, Sheetal Kumar Sahoo, Anil Raj, Narasimha Rao Guddala, Goldy Rathee

**Affiliations:** 1. Department of Pediatric and Preventive Dentistry, Hi-tech Dental College and Hospital, Health Park, Pandra, Rasulgarh, Bhubaneswar, India; 2. Department of Public Health Dentistry, Sarjug Dental College and Hospital, Darbhanga, Bihar, India; 3. Department of Prosthodontics, Vishnu Dental College, Bhimavaram, Andhra Pradesh, India; 4. Department of Conservative Dentistry and Endodontics, SGT Dental College, Gurugram, Haryana, India

**Keywords:** anxiety, CFSS-DS, dental dear, public health problem

## Abstract

In children, dental fear is not only associated with fear of pain or invasive procedures, but it is also correlated with separation from parents or confronting unfamiliar people or environments. The Children’s Fear Survey Schedule-Dental Subscale (CFSS-DS) was developed to evaluate dental fear in children, and this scale is now used across the world for evaluating dental fear. The aim of this study was to evaluate dental fear in children between 7–11 years of age and to find out the association between caries and fear of dental treatment. A total of 300 subjects of both sexes were enrolled in the study. Prior to the oral examination, all patients’ attendants were informed about the study, and the subjects were asked to fill in a questionnaire regarding the CFSS-DS scale. The data obtained through the questionnaires were analyzed using the Chi-Square test. Fear scores were highest for “injections” (3.91±0.17), “dentist drilling” (3.91±0.10) and “choking” (3.65±0.82). It was also observed that subjects who had already visited a dental clinic or those who were familiar with the dental environment at an early stage of life were less anxious than patients who were receiving dental treatment for the first time. In this study, we found that female subjects were more anxious in comparison to male patients. Once the child’s fear is identified, the dentist can use various behavior modification techniques to eliminate fear, explain the steps, and use the instruments accordingly until fear has vanished.

## Introduction

Fear is an unpleasant feeling caused by danger, which leads to metabolic or organ function changes, and it ultimately changes behavior [1, 2]. Dental fear is the fear of dentistry felt by the person who is receiving dental treatment from a health care professional [2]. Amongst all the fears, dental fear is ranked fourth, becoming a major public health problem [2]. The cause of dental fear in children is multifactorial as it can be related to one’s personality or previous painful experience, age, gender, parental dental fear, or general fear. Fears are usually mild, age-specific, and can go away as age increases. In contrast, a phobia is an extreme form of fear which persists throughout life. In children, dental fear hinders behavior management, which causes avoidance of dental situations and deterioration of oral hygiene. According to Kleinberg *et al.*, not all children who have dental fear show behavior management problems, and on the other hand, not all children showing behavior management problems have to be fearful [3]. Melamed Behavior Profile Rating Scale (1975) suggested that behavioral measurement techniques should be preferred over other behavioral measures. However, Hartman *et al.* concluded that behavioral measures might not be the best option for assessing dental fear [4]. Therefore, self-report questionnaires like Children’s Fear Survey Schedule-Dental Subscale (CFSS-DS), the one proposed by Cuthbert and Melamed in 1982 [5], and the Dental Anxiety Subscale (DAS) developed by Corah in 1969 are used since they are considered easy to administer and are statistically significant.

The CFSS-DS scale is a renowned psychometric scale to evaluate dental fear. In children, dental fear is a subjective experience, so assessment instruments entail disadvantages [5]. Therefore, fear questionnaires seem to be the best approach for ruling out dental fear. Literature shows that the CFSS-DS scale was preferred over the Venham Picture Test (VPT) proposed by Bengston and Cipes in 1977 and Corah’s DAS from 1969.

The aim of this cross-sectional study was:

• To assess dental fear in children aged 7–11 years old by using the CFSS-DS questionnaire;
• To describe the level of dental fear according to gender distribution.

## Material and Methods

A survey was conducted to assess dental fear in children who were undergoing dental treatment or already underwent dental treatment. A total of 300 children (both male and female) aged 7–11 years were randomly selected. Before recruiting participants for this study, permission was received from school authorities, and the study was explained in detail.

For this study, a close-ended questionnaire in both English and Hindi was administered. This questionnaire contained personal information and questions to evaluate the dental experience of children, a well as fear and anxiety related to it. The inclusion criteria were: children above 7 years of age who were not subjected to any invasive procedures but were aware of dental treatments. Children below 7 years of age, special needs children, or those who had already undergone some invasive procedures were excluded from this study. 

For this study, a very well-known dental fear scale – CFSS-DS – was used. It was created in 1982 to rule out dental fear in pediatric patients. Nowadays, this scale is used worldwide, and it has been translated into many languages so that it can be used globally. This questionnaire includes 10 questions with multiple choice answers and five different scores ranging from “low anxiety” (1) to “very afraid” (5). The total score could range from a minimum of 10 to a maximum of 50.

### Survey instrument

The CFSS-DS scale consists of 15 items related to unique aspects of dental care. The scores are rated as follows:

• Not Afraid = 1
• Little Afraid = 2
• Fairly Afraid = 3
• Quite Afraid = 4
• Very Afraid = 5

The total scores ranged from 15 to 75. Subjects with CFSS-DS scores greater than or equal to 38 were defined as dentally anxious, and they were placed in the “with dental fear” group, while those whose scores were less than 38 were included in the “without dental fear” group.

## Results

The study conducted was a closed-ended questionnaire study, which showed a high prevalence of dental anxiety, but severe anxiety/phobia was comparatively low. In this study, the CFSS-DS scale was used and scoring was assessed on the basis of a five-point Likert scale with five different scores ranging from “low anxiety” (1) to “very afraid” (5). Through this study, we found that many children were having a fear of “injections”, followed by “dental drilling machine”, then “instruments” and “fear of choking”, “hospital or clinic”, “doctor in a white uniform”, “stranger touch” and “fear from the dentist”. The data obtained through the questionnaires were analyzed using the Chi-Square test.

Table 1 depicts total children (both male and female) of different age groups ranging from 7–11 years and their CFSS-DS score.

**Table 1.** Total children of different Ages and the CFSS-DS Score.

Age (years)	**Male**	**Female**	**Frequency**	**CFSS-DS≥ 38**	**Percent**
**7**	32	28	60	35	20%
**8**	36	24	60	32	20%
**9**	27	33	60	27	20%
**10**	27	33	60	19	20%
**11**	31	29	60	10	20%
**Total**	153	147	300	123	100%

A total of 300 children were screened (both males and females). CFSS-DS scores ≥38 were identified in 123 children: 62 (40.50%) males and 61 (41.49%) females. A total of 177 children, out of which 91 (59.95%) were male and 86 (58.50%) female had CFSS-DS scores <38 (Table 2). There was no statistically significant difference between gender distribution and CFSS-DS scores (P>0.05), as seen in Table 3. The mean score for the CFSS-DS questionnaire is shown in Table 4 with no statistically significant difference in the mean score between boys and girls, except for questions 1 (P < 0.00), question 7 (P < 0.000), question 8 (p< 0.007) and question 14 (p <0.018) which were “stranger touch”, “having to go to the hospital”, “people in white uniform,” and “fear of the dentist”, respectively. Fear scores were highest for “injections” (3.91 ± 0.17), “dentist drilling” (3.91±0.10), and “choking” (3.65±0.82).

**Table 2.** Dental anxiety and its correlation with the sex of the patient.

**Sex**	**Number (%)**	**Dental fear (%)**
**Male**	153 (51%)	58 (47.1)
**Female**	147 (49%)	65 (52.8)
**Total**	300	123

**Table 3.** Gender distribution according to the CFSS-DS score.

**CFSS-DS**	**Male Number (Percentage)**	**Female Number (Percentage)**	**X^2^**	**P-value**
**<38**	82 (33.98%)	70 (46.61%)	0.000	0.876
**>38**	71 (46.40%)	77 (53.38%)	0.000	0.990
**Total**	153	147	-	-

**Table 4.** Comparison of mean CFSS-DS scores between males and females.

**Question Number**	**Males (Mean ± SD)**	**Females (Mean ± SD)**	**P-value**
**1**	2.45±0.68	3.21±0.47	0.000*
**2**	2.93±0.58	2.49±0.60	0.392
**3**	1.49±0.29	1.50±0.51	0.128
**4**	2.45±0.69	3.91±0.72	0.019
**5**	3.91±0.10	3.93±0.91	0.769
**6**	1.92±0.82	2.12±0.21	0.169
**7**	1.93±0.38	3.17±0.18	0.000*
**8**	1.45±0.72	2.45±0.37	0.007*
**9**	3.64±0.91	3.48±0.41	0.576
**10**	3.91±0.17	3.45±0.51	0.643
**11**	2.93±0.38	2.05±0.69	0.758
**12**	3.56±0.01	3.67±0.16	0.286
**13**	3.65±0.82	3.49±0.83	0.732
**14**	1.49±0.91	2.83±0.42	0.018*
**15**	2.10±0.62	1.29±0.28	0.932

*p<0.05

## Discussion

Dental treatment usually induce fear in children. So, to prevent this anxiety, it is essential to identify anxious patients at an early age. In India, we have noticed that many people consider dental treatment as “treatment of negligence” until and unless they face some dental emergencies [6], which further increases their fear of dentistry [7]. Literature shows that 50% of individuals develop extremely high dental fear during childhood, while 27% during adolescence and 23% during adulthood [8].

We used the CFSS-DS scale in this study because it is globally used and it is convenient to use in pediatric patients. It also has good reliability and validity to assess anxiety levels [9, 10]. Usually, child behavior rating is assessed in dental clinics [11], but the anxiety level is not evaluated regularly. It has also been noted that anxiety is assessed by various psychometric scales [12], but anxiety’s trigger was not properly recorded until now. Therefore, by using the CFSS-DS scale, which records fear and anxiety, we found that fear and anxiety are most likely to happen by injection, dental drilling, or choking.

To date, several measurement techniques such as psychological scales, behavioral rating, and psychometric techniques have been used to find out dental behavior management problems [12–15].

Many studies have been conducted on this theme globally. In the current study, the CFSS-DS score was 28.5, which resembles a previous study that was conducted in India [16], and it was significantly higher than other country scores like Sweden (23.1) [3], Japan (24.6) [9] and Netherlands (23.9) [10]. However, the score was comparatively lower in Singapore (30.6) [7], Canada (for Chinese children – 31.9), and China (35) [17]. 

Atram *et al.* reported that 13% of patients experience fear of dental instruments [18]. In our study, we also found that individuals feared dental instruments along with choking, while several individuals also reported a fear of injection.

Therefore, in order to overcome the level of dental fear among children, it is necessary to assess the clinical risk by using certain epidemiological concepts to implicate preventive treatments like pit and fissure sealant, fluoride application, routine oral health examinations, oral hygiene instructions, or to conduct school dental health programs or caries activity tests. Also, it is extremely important to educate parents about preventive measures that can be taken at early ages, reducing the need for injection and further treatments [19].

Dental anxiety is a major problem that has a direct influence on the oral hygiene of children and adults. Hence, it is important to detect the cause of fear and break this vicious cycle of dental fear, developing a more optimistic attitude towards dental treatments, ultimately leading to good oral hygiene.

Although CFSS-DS is a reliable questionnaire to assess dental fear amongst children, further studies should be carried out on a large population to correlate the CFSS-DS score to the actual behavioral observations in the dental clinic.

## Conclusion

This survey study showed that dental fear was 47.15% in males and 52.8% in females in children aged between 7-11 years old. It was found that most children were having a fear of “injections”, followed by “dental drilling machine”, then “instruments” and “fear of choking,” “hospital or clinic”, “doctor in a white uniform”, “stranger touch” and “fear from the dentist”. Therefore, once the fear of a child is identified, the dentist can use various behavior modification techniques and instill a positive dental attitude towards the future dental procedure.

## Acknowledgements

### Conflict of interest

The authors declare that there is no conflict of interest.

### Ethical approval

Ethical approval for this study was obtained from the Hi-Tech Dental College Ethical Board (approval no. 126/10-2017).

### Informed consent

All subjects agreed to participate in the study and written informed consent was obtained from school authorities.
